# Comparison of the prevalence of antimicrobial-resistant *Escherichia coli*, verotoxin-producing *E. coli* and enteropathogenic *E. coli* in griffon vultures (*Gyps fulvus*), cinereous vultures (*Aegypius monachus*) and red kites (*Milvus milvus*) fed in the wild and in a rescue centre

**DOI:** 10.3389/fvets.2025.1601149

**Published:** 2025-06-09

**Authors:** Alejandra Cerezo-Caro, Alicia Mas, Fernando González-González, Andrea Rodríguez-Carrión, Saskia C. Flament-Simon, Jesús E. Blanco, Abel Martínez-Rodrigo, Clara Hurtado-Morillas, Gustavo Domínguez-Bernal, José A. Orden

**Affiliations:** ^1^Seashore Environment and Fauna, Cádiz, Spain; ^2^Wildlife Hospital, Group for the Rehabilitation of Native Fauna and its Habitat (GREFA), Madrid, Spain; ^3^INMIVET, Department of Animal Health, School of Veterinary Medicine, Complutense University of Madrid, Madrid, Spain; ^4^Departmental Section of Pharmacology and Toxicology, School of Veterinary Medicine, Complutense University of Madrid, Madrid, Spain; ^5^Clínica Veterinaria Dogos, Madrid, Spain; ^6^Escherichia coli Reference Laboratory (LREC), School of Veterinary Medicine, University of Santiago de Compostela, Lugo, Spain; ^7^INMIVET, Department of Animal Science, School of Veterinary Medicine, Complutense University of Madrid, Madrid, Spain; ^8^Centro de Investigación en Sanidad Animal, Instituto Nacional de Investigación y Tecnología Agraria y Alimentaria, Consejo Superior de Investigaciones Científicas (CISA-INIA-CSIC), Madrid, Spain

**Keywords:** vultures and red kites, antimicrobial-resistant *E. coli*, verotoxin-producing *E. coli* (VTEC), enteropathogenic *E. coli* (EPEC), One Health

## Abstract

**Introduction:**

Antimicrobial resistance in *Escherichia coli* from avian scavengers remains poorly characterized, with limited data available for griffon vultures (*Gyps fulvus*) and no studies on cinereous vultures (*Aegypius monachus*) or red kites (*Milvus milvus*). In addition, the presence of verotoxin-producing *E. coli* (VTEC) and enteropathogenic *E. coli* (EPEC), both zoonotic pathogens, in these animal species has not been studied before.

**Methods:**

A total of 282 *E. coli* isolates were recovered from faecal samples of 28 griffon vultures, 22 cinereous vultures and 13 red kites. Isolates were tested for resistance to 14 antimicrobial agents and screened for *vt1*, *vt2*, and *eae* genes. Sampling was performed upon arrival at a wildlife rescue centre and after several weeks of housing that centre.

**Results:**

High levels of antimicrobial resistance (25–50%) were detected for amoxicillin-clavulanic acid, ceftriaxone, tetracycline, trimethoprim-sulphamethoxazole and nalidixic acid, and very high (>50%) for ampicillin, streptomycin, kanamycin, amikacin, gentamicin, sulphafurazole and ciprofloxacin. No significant differences in antimicrobial resistance prevalence were observed between initial and follow-up samplings. In addition, two VTEC isolates were detected in a cinereous vulture, and five EPEC isolates were identified in a griffon vulture and four cinereous vultures. All VTEC and EPEC isolates were detected in a single sampling event.

**Conclusion:**

These findings indicate that vultures and red kites are an important reservoir of antimicrobial-resistant *E. coli*. Measures should be implemented to minimize their exposure to antimicrobials or antimicrobial-resistant bacteria in both natural environments and rescue centres. Furthermore, the detection of VTEC and EPEC suggests that vultures may act as occasional carriers of zoonotic *E. coli*, highlighting potential public health concerns.

## 1 Introduction

The griffon vulture (*Gyps fulvus*) and the cinereous vulture (*Aegypius monachus*) are obligate scavengers (1, 2), while the red kite (*Milvus milvus*) is an opportunistic predator and facultative scavenger (3). In Europe, adult griffon vultures are resident and show high fidelity to their breeding colonies, while a substantial fraction of juveniles are migratory and overwinter in Africa at least during their first year of life. The Iberian Peninsula hosts the largest population of griffon vultures on the European continent, with 90% of all breeding pairs (2). The cinereous vulture is the biggest raptor in Europe and its range is irregularly extended from the Iberian Peninsula to Asia (1). In Spain, there have been estimated 43 colonies and 2,500 breeding pairs of cinereous vultures (around 20% of the individuals worldwide) and the Iberian population is considered mainly resident, but some juveniles seldom migrate to western Africa (4). The red kite is mostly distributed in Europe and Spain holds one of the most abundant breeding populations and represents its main stronghold as a wintering area, with around 50,000 individuals (3).

Antimicrobial resistance is a global issue of concern and a critical One Health challenge, driven by the interconnectedness of human, animal and environmental health (5, 6). Commensal bacteria constitute a reservoir of resistance genes for potentially pathogenic bacteria. Their level of resistance is a good indicator for selection pressure by antimicrobial use and for resistance problems to be expected in pathogens. Monitoring the prevalence of antimicrobial resistance in indicator bacteria, such as faecal *Escherichia coli*, in different populations, animals, patients and healthy humans makes it feasible to compare the prevalence of antimicrobial resistance and to detect the transfer of resistant bacteria or resistance genes from animals to humans and vice versa (7). The prevalence of antimicrobial-resistant bacteria harboured by wildlife generally appears to be primarily influenced by the relative level of exposure to anthropogenic antimicrobial resistance contamination and through associated selection pressures imparted within the environment (8). As such, wildlife may be good indicators of the burden of antimicrobial resistance within the local environment and may therefore be useful for identifying potential point sources of anthropogenic antimicrobial resistance contamination (8). In addition, resident and migratory wild birds may play a key role in spreading antimicrobial resistance by acquiring resistant bacteria and dispersing them through the environment (9). In this context, the emergence and spread of antimicrobial resistance in avian scavengers have become a growing concern (10, 11).

*E. coli* is a commensal bacterium found in the intestinal tracts of humans and many animals, and it may constitute a reservoir of antimicrobial resistance genes for pathogenic bacteria (7). In contrast to *E. coli* from domestic and food-producing animals, there is little information regarding the antimicrobial resistance of bacteria isolated from wild animals (12). Thus, only a few studies have investigated the frequency of antimicrobial resistance in *E. coli* from different species of vultures, including griffon vultures (6, 10, 11, 13–15), and, to our knowledge, none have focused on *E. coli* from cinereous vultures or red kites. In Spain, vultures primarily obtain their food from pig and poultry carcasses from factory farms, which are disposed of in supplementary feeding stations, a major source of antimicrobial exposure in their breeding areas (10). Additionally, landfills act as hotspots for the acquisition of antimicrobial-resistant bacteria of human origin (16). On the other hand, red kites in Spain have a highly opportunistic diet, predominantly feeding on wild rabbits, particularly those affected by diseases. During times of food scarcity, they often rely on landfills, roadkill and livestock carcasses at feeding stations (3, 17). In addition, wild birds housed in rehabilitation centres are usually fed with foods from different animal sources, which may contain high levels of resistant bacteria, as well as resistance genes (18).

In humans, infections with verotoxin (VT)-producing *E. coli* (VTEC), also called Shiga toxin-producing *E. coli*, cause illnesses ranging from mild diarrhoea to haemorrhagic colitis and haemolytic uremic syndrome. Domestic ruminants, mainly cattle, have been implicated as the principal reservoir of these strains for humans (19). However, VTEC have also been isolated from wild animals (5, 20–27). In addition to VT, VTEC can synthesize the adhesin intimin (encoded by the *eae* gene). Enteropathogenic *E. coli* (EPEC) strains possess the *eae* gene but do not produce VT. EPEC have been classified as typical (possessing the *bfpA* gene) or atypical (lacking the *bfpA* gene). Typical EPEC (tEPEC) are a major cause of infantile diarrhoea in developing countries and are found, with few exceptions, only in humans. Atypical EPEC (aEPEC) are associated with diarrhoea in both developing and developed countries, and can be isolated from humans and animals, including wildlife (19, 20, 23, 27–29). In contrast with other wildlife, the presence of VTEC and EPEC in vultures and kites has not been previously studied.

The main objective of this study was to assess the prevalence of antimicrobial-resistant *E. coli*, VTEC and EPEC isolates in griffon and cinereous vultures and red kites in Spain. Additionally, this study aimed to evaluate the influence of different factors, such as diet and population density, on the prevalence of antimicrobial resistant *E. coli*, VTEC and EPEC isolates found in these avian scavengers.

## 2 Materials and methods

### 2.1 Animals

From June 2021 to May 2022, all griffon vultures, cinereous vultures and red kites admitted at the Wildlife Rescue Centre (WRC) managed by Grupo de Rehabilitación de la Fauna Autóctona y su Hábitat (GREFA) were examined and sampled. GREFA, based in the Madrid region (central Spain), admits more than 7,000 wild animals each year. These include various species of birds, mammals and reptiles from Iberian fauna, with the aim of rehabilitating and releasing them back into their natural habitat. The main reasons of admission to the WRC are related to human activities, such as hunting, accidents with power lines (electrocution or traumas), and collisions with windows or cars, among others. Additionally, natural diseases of wildlife are another cause of admission. During the initial examination of the birds included in this study, a cloacal swab was taken from each animal for *E. coli* isolation, prior to any treatment. At the WRC, griffon vultures and cinereus vultures were provided with a diet consisting of rabbits, chickens, and bovine hearts, all sourced from authorized slaughterhouses. On the other hand, red kites were fed day-old chicks acquired from a commercial provider specialized in raptor feed. The WRC’s water supply came from the municipal potable water system. When it was possible, animals were sampled up to two more times (the second sampling was carried out at least 1 month after the admission of the animal, and the third at least 1 month after the second sampling). Samples were preserved in a transport medium with activated charcoal at 4°C and processed within seven days of collection. Information about species, area of origin, and clinical data from each animal was recorded when possible.

Although the study included birds from eight different Spanish regions, most of them came from the Madrid region. According to their origin, three different areas within the Madrid region were established to assess potential geographical differences associated to population density: rural (<150 inhabitants/km^2^), periurban (150–1,000 inhabitants/km^2^) and urban (>1,000 inhabitants/km^2^).

### 2.2 Isolation of *Escherichia coli*

Faecal samples were plated on MacConkey agar to isolate *E. coli*. After overnight incubation, up to three colonies with the typical appearance of *E. coli* were randomly selected from each sample. Isolates were identified as *E. coli* by biochemical tests, including catalase, oxidase, indole, methyl-red Voges–Proskauer, citrate and urease.

### 2.3 Antimicrobial susceptibility of *Escherichia coli*

Antimicrobial testing was performed using the disc diffusion method, following the recommendations of the Clinical and Laboratory Standards Institute (CLSI, 2022) (30). The growth inhibition area of each isolate was measured, and then each isolate was classified as susceptible, intermediate, or resistant based on the breakpoints provided by the CLSI (2022) (30). The following 14 antimicrobials, belonging to six different classes, were tested: ampicillin, amoxicillin-clavulanic acid, cefoxitin, and ceftriaxone (β-lactams); streptomycin, kanamycin, amikacin, and gentamicin (aminoglycosides); tetracycline (tetracyclines); chloramphenicol (phenicols); sulphafurazole and trimethoprim-sulphamethoxazole (inhibitors of the folic acid pathway); and nalidixic acid and ciprofloxacin (quinolones). All antimicrobial susceptibility discs were provided by Oxoid, Thermo Fisher Scientific, Waltham, Massachusetts, United States. *E. coli* ATCC 25922 was used as a control strain.

### 2.4 Detection and characterization of VTEC and EPEC isolates

All the *E. coli* isolates were tested using PCR for the presence of the *vt1*, *vt2*, *eae*, and *bfpA* genes as described previously (23).

### 2.5 Serotyping

VTEC and EPEC isolates were serotyped using the agglutination method as described previously (28) with all available O (O1-O185) and H (H1-H56) antisera.

### 2.6 Statistical analysis

Statistical analyses were performed using the Shapiro–Wilk normality test to assess the normal distribution of quantitative variables. The percentage of antimicrobial resistance of *E. coli* isolates from the first sampling in each of the species studied was compared using the one-way analysis of variance (ANOVA) with Tukey’s *post hoc* test for multiple comparisons to determine which means from the independent groups were significantly different. When analysing the percentage of antimicrobial resistance of *E. coli* isolates in all different samplings among the species, Kruskal–Wallis test was employed, as these data did not follow a normal distribution. All analyses were performed using GraphPad Prism software (version 8.3.0 for Windows, San Diego, California, United States). Significant differences were determined and are designated with asterisks, as follows: ^*^p < 0.05 and ^**^p < 0.01.

## 3 Results

### 3.1 Isolation and antimicrobial susceptibility of *Escherichia coli*

A total of 80 animals were sampled, including 33 griffon vultures, 25 cinereous vultures and 22 red kites. However, not all animals could be sampled for a second and/or third time due to their poor condition upon arrival and subsequent lack of improvement. Additionally, not all samples yielded three *E. coli* isolates and some yielded none. As a result, altogether, 282 isolates from 63 birds (78.8%) were identified as *E. coli*: 123 from 28 (84.8%) griffon vultures, 120 from 22 (88%) cinereous vultures, and 39 from 13 (59.1%) red kites. Table 1 shows the distribution of *E. coli* isolates by animal species and sampling number. Seventy-one *E. coli* isolates were found in 28 animals (11 griffon vultures, 12 cinereous vultures and 5 red kites) from the Madrid region in the first sampling. Table 2 shows the number of *E. coli* isolates found in the Madrid region in the first sampling by animal species and areas classified by population density.

**Table 1:** Number of *E. coli* isolates by animal species and sampling number.

Animal species	First sampling	Second sampling	Third sampling	Total
Red kites	11	23	5	39
Griffon vultures	55	62	6	123
Cinereous vultures	49	59	12	120

**Table 2:** Number of *E. coli* isolates found in the Madrid region in the first sampling by animal species and areas classified by population density.

Animal species	Rural	Periurban	Urban	Total
Red kites	0	7	4	11
Griffon vultures	19	10	0	29
Cinereous vultures	6	7	18	31

Although the studied animals had no previous antibiotic treatments, the results showed that all *E. coli* isolates, but one, were resistant to at least one antimicrobial. The number and percentage of *E. coli* isolates from griffon vultures, cinereous vultures and red kites resistant to the antimicrobials studied are presented in Table 3. Overall, the antimicrobial resistance percentages were high (25–50%) for amoxicillin-clavulanic acid, ceftriaxone, tetracycline, trimethoprim-sulphamethoxazole and nalidixic acid, and very high (>50%) for ampicillin, streptomycin, kanamycin, amikacin, gentamicin, sulphafurazole and ciprofloxacin. In contrast, 9.9 and 12.8% of the *E. coli* isolates were resistant to cefoxitin and chloramphenicol, respectively. Nine of the 14 antimicrobials tested (ampicillin, amoxicillin-clavulanic acid, ceftriaxone, streptomycin, kanamycin, amikacin, gentamicin, nalidixic acid and ciprofloxacin) are considered critically important antimicrobials for human medicine (31), and in all cases the observed level of antimicrobial resistance was high or very high (Table 3).

**Table 3:** Number and percentage of *E. coli* isolates from red kites, griffon vultures and cinereous vultures resistant to the antimicrobials studied.

Animal species (*n*)	AMP^a^	AMC^a^	FOX	CRO^a^	S^a^	K^a^	AK^a^	CN^a^	TE	C	SF	SXT	NA^a^	CIP^a^
Red kites (*n* = 39)	19 (48.7)	9 (23.1)	0 (0.0)	4 (10.2)	36 (92.3)	26 (66.6)	25 (64.1)	29 (74.4)	11 (28.2)	8 (20.5)	30 (76.9)	9 (23.1)	8 (20.5)	15 (38.5)
Griffon vultures (*n* = 123)	75 (61.0)	42 (34.1)	9 (7.3)	28 (22.8)	103 (83.7)	86 (69.9)	82 (66.7)	79 (64.2)	56 (45.5)	16 (13.0)	107 (87.0)	33 (26.8)	35 (28.5)	45 (36.6)
Cinereous vultures (*n* = 120)	109 (90.8)	84 (70.0)	19 (15.8)	83 (69.2)	101 (84.2)	108 (90.0)	99 (82.5)	106 (88.3)	42 (35.0)	12 (10.0)	116 (96.7)	57 (47.5)	74 (61.7)	95 (79.2)
Total (*n* = 282)	203 (72.0)	135 (47.9)	28 (9.9)	115 (40.8)	240 (85.1)	220 (78.0)	206 (73.0)	214 (75.9)	109 (38.6)	36 (12.8)	253 (89.7)	99 (35.1)	117 (41.5)	155 (55.0)

AMP, ampicillin; AMC, amoxicillin-clavulanic acid; FOX, cefoxitin; CRO, ceftriaxone; S, streptomycin; K, kanamycin; AK, amikacin; CN, gentamicin; TE, tetracycline; C, chloramphenicol; SF, sulphafurazole; SXT, trimethoprim-sulphamethoxazole; NA, nalidixic acid; CIP, ciprofloxacin.

a Antimicrobials considered as critically important for human medicine (31).

Most *E. coli* isolates showed multiclass resistance: 35 isolates were resistant to two antimicrobial classes, 55 isolates to three antimicrobial classes, 114 isolates to four antimicrobial classes, 42 isolates to five antimicrobial classes and 25 isolates to six antimicrobial classes.

**Figure 1:**
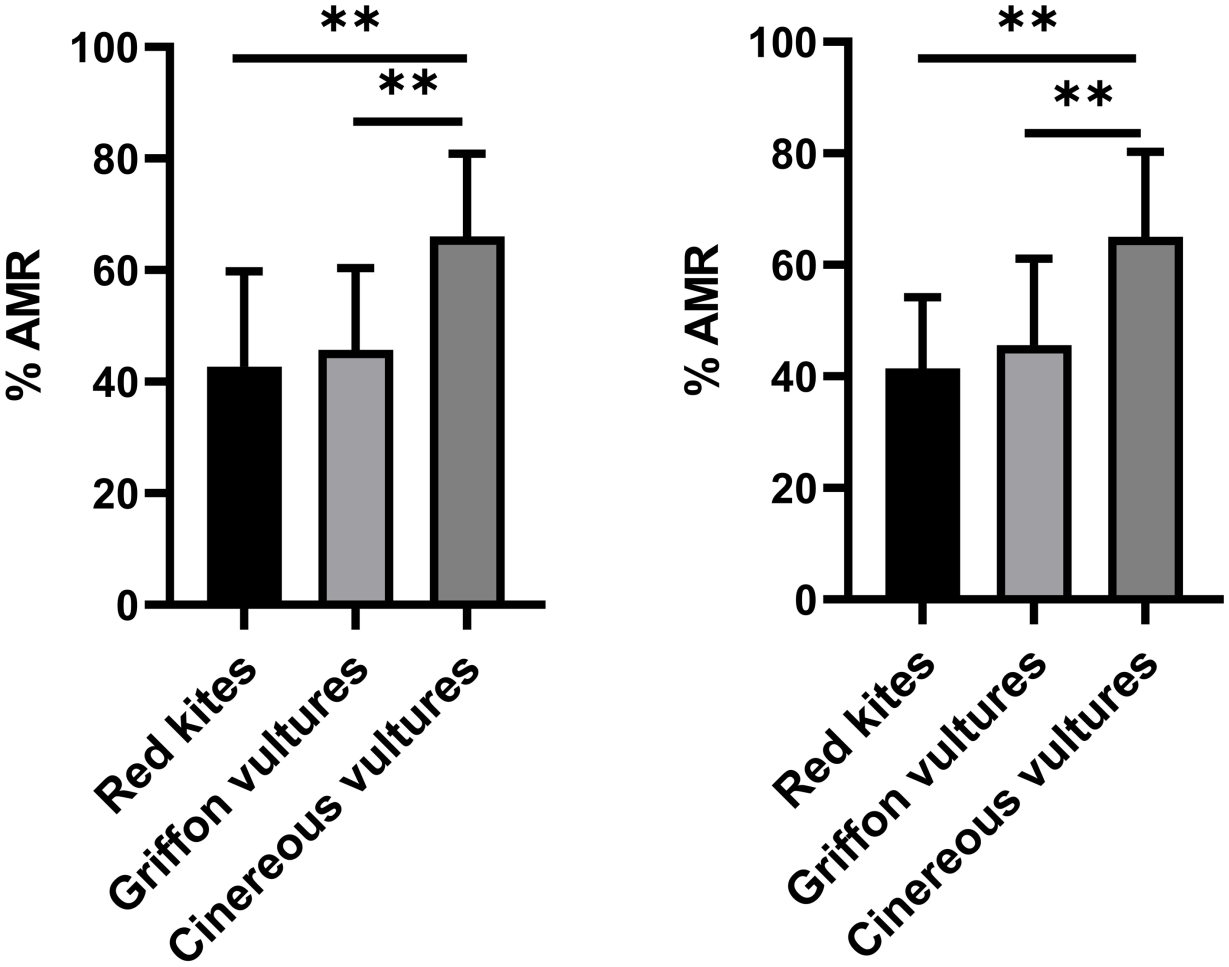
Comparison of the average percentage of total *E. coli* isolates (left) and *E. coli* isolates found in the first sampling (right) resistant to antimicrobials in red kites, griffon vultures, and cinereous vultures. Data are represented as the mean ± SD. Asterisks (*) indicate statistically significant differences (*p < 0.05, **p < 0.01).

The average percentage of total *E. coli* isolates resistant to antimicrobials in cinereous vultures (66.0%, IC95, 38.1–97.6%) was significantly higher than those found in griffon vultures (45.7%, IC95, 19.0–85.7%) and red kites (42.7%, IC95, 21.43–78.6%) (Figure 1). These differences were also observed when comparing the average percentages of antimicrobial-resistant *E. coli* isolates in the three animal species studied in the first sampling (on the day the animals entered the WRC) (Figure 1).

**Figure 2:**
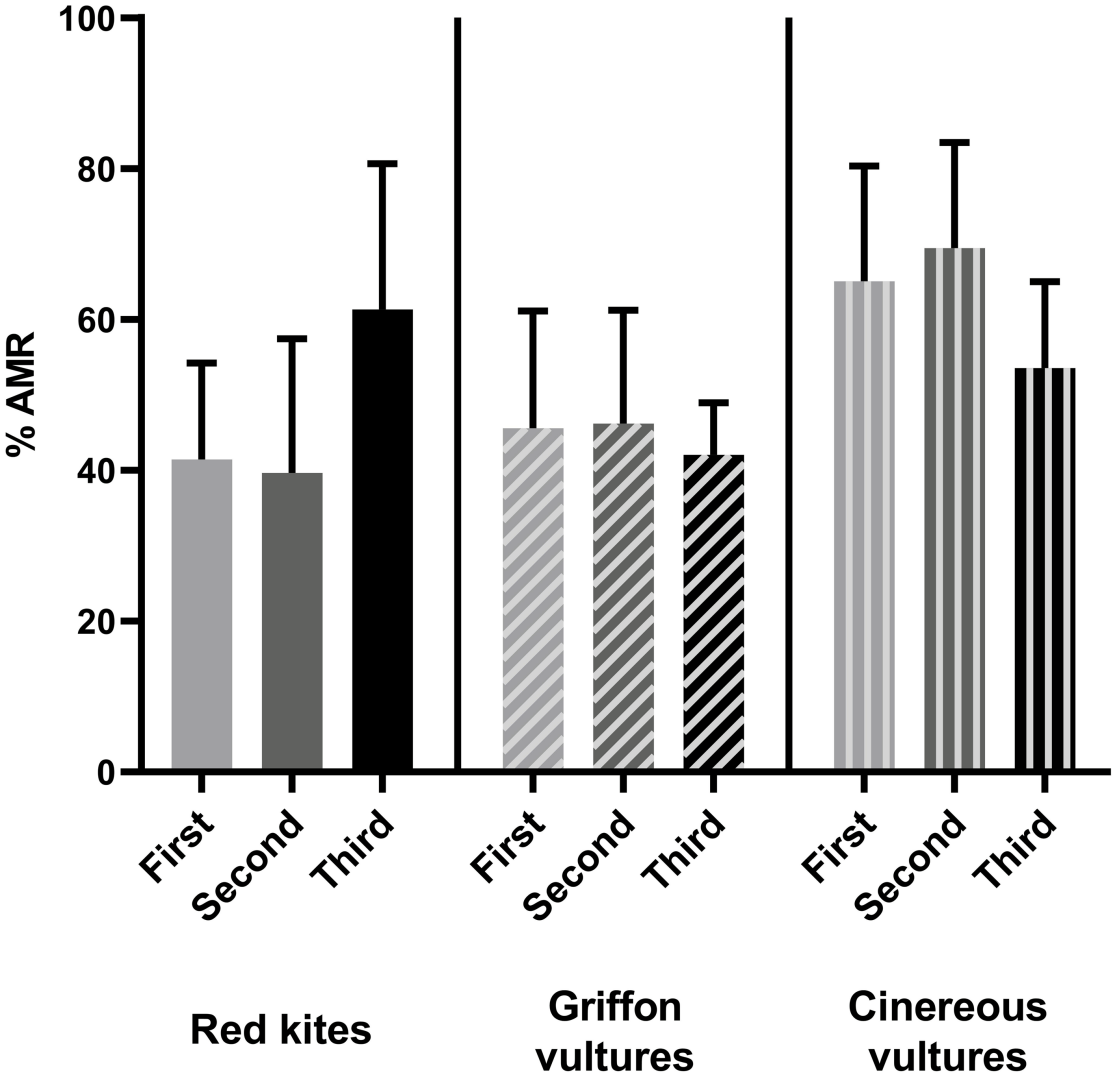
Comparison of the mean percentage of antimicrobial resistance of *E. coli* isolates from the first sampling with those from the second sampling and from the second sampling with those from the third sampling in red kites, griffon vultures and cinereous vultures. Data are represented as the mean ± SD.

**Figure 3:**
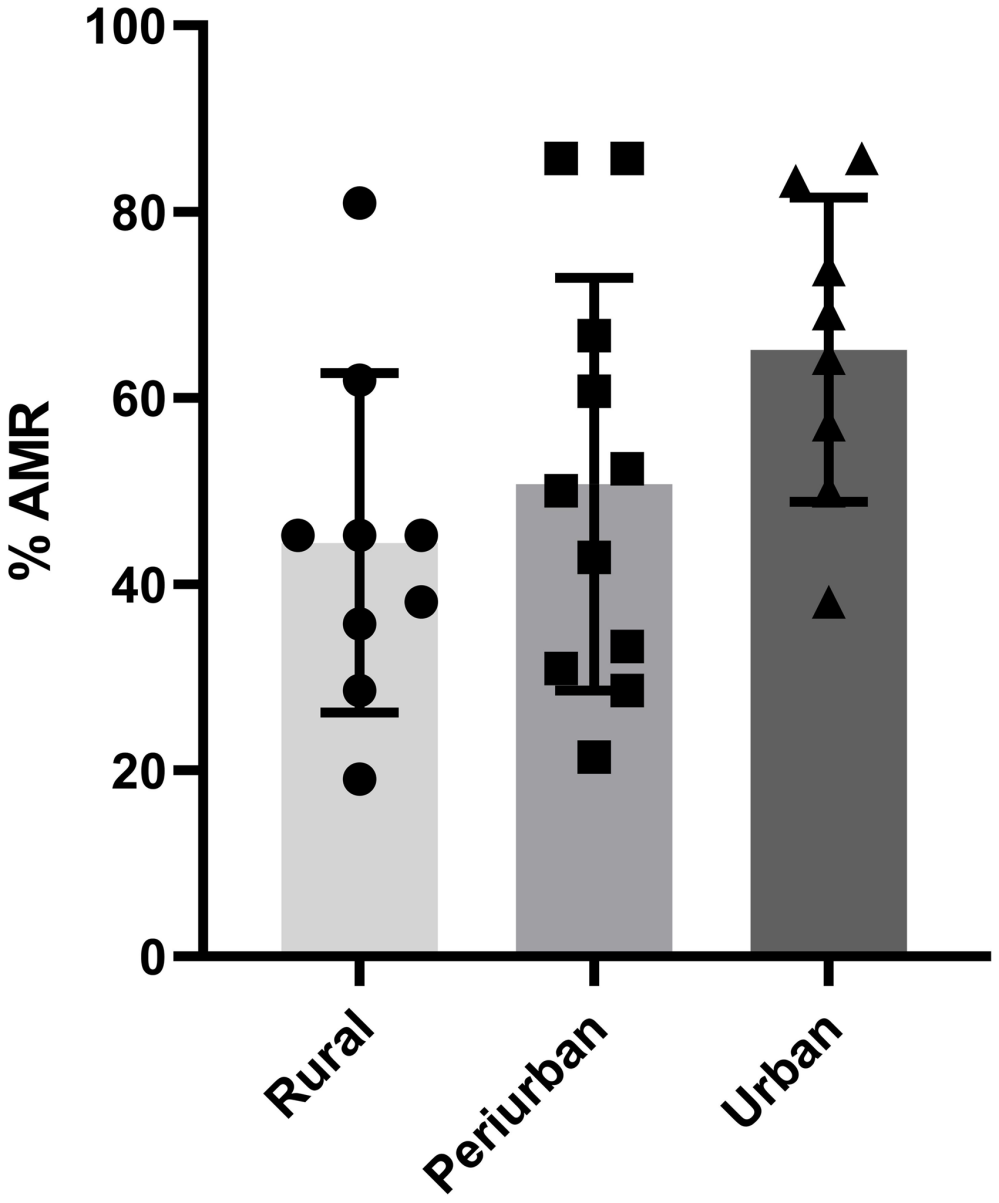
Comparison of the mean percentage of antimicrobial resistance in the first sampling of *E. coli* isolates from red kites, griffon vultures and cinereous vultures from the Madrid region of the three areas classified by population density. Data are represented as the mean ± SD.

No statistically significant differences were found when comparing the mean frequencies of antimicrobial resistance of *E. coli* isolates from the first sampling with those from the second sampling, or when those frequencies from the second sampling were compared with those from the third sampling, in any of the animal species studied (Figure 2). Similarly, no statistically significant differences were found when comparing the mean frequencies of antimicrobial resistance in *E. coli* isolates from the first sampling in red kites and vultures from the Madrid region across the three areas classified by population density (Figure 3).

### 3.2 Detection and characterization of VTEC and EPEC isolates

Two VTEC isolates were found in one of the 80 (1.3%) animals studied. These VTEC isolates were detected during the first sampling of a cinereous vulture and were positive to *vt2*, negative to *eae* and belonged to the same serotype (O174:H21), although they showed different antimicrobial resistance profiles (Table 4). In addition, five EPEC isolates were detected in the cloacal samples of five of the 80 (6.3%) birds studied (one griffon vulture and four cinereous vultures). These EPEC isolates were classified as aEPEC (positive to *eae* but negative to *vt* and *bfpA*) and belonged to five different serotypes (Table 4).

**Table 4:** Characteristics of VTEC and aEPEC isolates detected in the faeces of griffon and cinereous vultures.

Isolate reference	Animal reference	Origen (region) of the animal	No. of sampling	Pathotype	Serotype	*vt* type	Resistance phenotype
GV6D	GV6	Madrid	Second	aEPEC	O80:H-	—	S, K, TE, SF, NA, CIP
CV2E	CV2	Madrid	Second	aEPEC	O2:H40	—	AMP, AMC, CRO, S, K, AK, CN, SF, SXT, NA, CIP
CV3A	CV3	Madrid	First	aEPEC	O177:H-	—	AMP, CRO, S, K, AK, CN, SF, SXT, CIP
CV5A	CV5	Madrid	First	VTEC	O174:H21	*vt2*	AMP, AMC, CRO, S, K, AK, CN, SF, CIP
CV5C	CV5	Madrid	First	VTEC	O174:H21	*vt2*	AMP, SF, CIP
CV9E	CV9	La Rioja	Second	aEPEC	O40:H10	—	AMP, AMC, CRO, K, AK, CN, SF, CIP
CV12G	CV12	Madrid	Third	aEPEC	ONT:H-	—	AMP, AMC, CRO, S, K, AK, CN, SF, NA, CIP

GV, griffon vulture; CV, cinereous vulture; NT, not typeable; AMP, ampicillin; AMC, amoxicillin-clavulanic acid; CRO, ceftriaxone; S, streptomycin; K, kanamycin; AK, amikacin; CN, gentamicin; TE, tetracycline; SF, sulphafurazole; SXT, trimethoprim-sulphamethoxazole; NA, nalidixic acid; CIP, ciprofloxacin.

## 4 Discussion

From a One Health perspective, it is essential to study the role of wildlife in the persistence and dissemination of zoonotic and antimicrobial-resistant bacteria. In this context, this study provides, for the first time, a longitudinal analysis of the antimicrobial-resistant *E. coli,* VTEC and EPEC isolates in vultures and red kites in Spain.

The high levels of antimicrobial resistance observed in *E. coli* isolates from the first sampling likely reflected the exposure of avian scavengers to resistant bacteria from carcasses of medicated livestock disposed at supplementary feeding stations or from human waste in landfills (10, 16, 32). The differences in the antimicrobial resistance among cinereous vultures, griffon vultures and red kites found in the first sampling may be due to host factors. Thus, some host factors, such as diet, may affect the dynamics of gut microbiota and, therefore, the prevalence of resistant bacteria among commensal gut bacteria (12, 33). The higher average percentage of antimicrobial-resistant *E. coli* isolates in the first sampling in cinereous vultures compared to red kites could be partly attributed to the fact that cinereous vultures in Spain mainly feed at supplementary feeding stations or landfills (10, 16), while the main source of food for red kites are wild rabbits and roadkill, and only occasionally scavenge at landfills (3, 17). However, the reasons for the differences in antimicrobial resistance between cinereous vultures and griffon vultures in the first sampling remain unclear and warrant further investigation.

No statistically significant differences were observed in the prevalence of antimicrobial resistance between *E. coli* isolates from the first sampling compared to those from the second sampling, nor when frequencies from the second sampling were compared with those from the third sampling. However, vultures and kites do not seem to be reservoirs of antimicrobial-resistant *E. coli* isolates, since in the 37 animals in which *E. coli* isolates were found in more than one sampling, the antimicrobial resistance profiles of the *E. coli* isolates from the different samples of the same animal were, except for one, always different. This suggests that vultures and kites are likely acquiring antimicrobial-resistant *E. coli* from their environment rather than maintaining them. To test this hypothesis, it would be interesting to conduct additional longitudinal studies on these birds, including longitudinal studies in natural environments.

The detection of high antimicrobial resistance in *E. coli* isolates from the second and third samplings suggests a considerable risk of spreading resistant bacteria through the food chain. This may occur due to bacteria derived from food-producing animals or through cross-contamination during food processing. Supporting this hypothesis, Pinto et al. (18) found that raw food may be an important source of multi-resistant *E. coli* for wild birds kept in rehabilitation centres and recommended developing regulations regarding food sources provided to animals housed in wildlife rescue centres. Water has also been identified as a potential source of antimicrobial resistance. Although typically considered low-risk, drinking water may act as a reservoir for antimicrobial-resistant bacteria, such as *E. coli*, as well as antimicrobial resistance genes (34, 35). Moreover, direct contact with other interned animals and handlers may also contribute to the transmission of antimicrobial-resistant bacteria in rehabilitation centres. Furthermore, sick birds entering wildlife rescue centres may suffer from gut dysbiosis and weakened immune defences, which could make them more vulnerable to intestinal colonization by new bacterial strains (18). These findings should also be considered when establishing animal handling guidelines to reduce the risk for workers in animal rescue centres and to prevent the contamination of natural habitats after the release of birds.

Particularly worrisome is the high prevalence of resistance, above 40%, against nine antimicrobials of critical importance for human medicine. The percentages of *E. coli* isolates from griffon vultures resistant to the antimicrobials used in this study were generally higher than those previously reported for *E. coli* isolated from that vulture species in Spain (10, 11, 13). However, some studies showed similar resistance frequencies to ampicillin (11, 13) and amoxicillin-clavulanic acid (10), while the frequencies of resistance to chloramphenicol (10, 11), tetracycline (10, 11), and trimethoprim-sulphamethoxazole (11, 13) were higher than those found in griffon vultures in this study.

No statistically significant differences were found when comparing the mean frequencies of antimicrobial resistance in the first sampling of *E. coli* isolates from vultures and red kites from rural, periurban and urban areas in the Madrid region. In agreement with our results, Jardine et al. (36) reported no difference in the prevalence of antimicrobial-resistant *E. coli* in raccoon faecal samples from rural and urban areas in Ontario, Canada.

To our knowledge, this study is the first report of the presence of VTEC and EPEC in vultures. Only one and five vultures carried VTEC and EPEC isolates, respectively, in their cloaca. Other studies conducted in wild birds have found similar prevalence rates of VTEC (0–1.3%) (5, 20, 37), but slightly lower prevalence rates of EPEC (1.3–4.9%) (20, 37, 38). All EPEC isolates from vultures were classified as aEPEC, which is consistent with previous studies showing that most of EPEC isolates from livestock and wild animals are aEPEC (19, 20, 23, 38). Four of the five aEPEC isolates detected in this study were found in the second or third sampling, demonstrating that vultures generally acquired aEPEC at the rescue center. In addition, all VTEC and EPEC isolates found in this study were detected in a single sampling event. In contrast, longitudinal studies performed on domestic ruminants have shown that these species are persistent VTEC shedders (39). Overall, our results suggest that vultures are occasional carriers of VTEC and EPEC, probably because these *E. coli* pathotypes are not well adapted to colonize the intestine of these hosts, and therefore vultures may be considered only a limited hazard to human health by transmission of VTEC and EPEC to the environment.

## 5 Conclusion

Griffon and cinereous vultures and red kites are common carriers of multi-resistant *E. coli* isolates and, consequently, they may represent a considerable hazard to human and animal health by transmitting these isolates to food and environment through their faeces. However, these species are only occasional carriers of VTEC and EPEC. Our study underscores the need for measures to reduce the exposition of avian scavengers to antimicrobials or antimicrobial-resistant bacteria at supplementary feeding stations and landfills. In addition, to prevent the spread of resistant bacteria through the food chain in wild birds housed in wildlife rescue centres, it is necessary to avoid providing raw meat from food-producing animals and to adopt effective hygiene procedures to minimize cross-contamination between different foods.

## Data Availability

The raw data supporting the conclusions of this article will be made available by the authors, without undue reservation.

## Supplementary material

SUPPLEMENTARY TABLE S1 Antibiotic resistance profiles of *E. coli* isolates from red kites, griffon vultures and cinereous vultures.
